# Skeletal muscle wasting with disuse atrophy is multi-dimensional: the response and interaction of myonuclei, satellite cells and signaling pathways

**DOI:** 10.3389/fphys.2014.00099

**Published:** 2014-03-17

**Authors:** Naomi E. Brooks, Kathryn H. Myburgh

**Affiliations:** ^1^Health and Exercise Science Research Group, School of Sport, University of StirlingStirling, UK; ^2^Muscle Research Group, Department of Physiological Sciences, Stellenbosch UniversityStellenbosch, South Africa

**Keywords:** skeletal muscle atrophy, muscle cell signaling, myostatin, MuRF1, MAFbx, IGF1-AKT-mTOR, unloading, resistance exercise

## Abstract

Maintenance of skeletal muscle is essential for health and survival. There are marked losses of skeletal muscle mass as well as strength and physiological function under conditions of low mechanical load, such as space flight, as well as ground based models such as bed rest, immobilization, disuse, and various animal models. Disuse atrophy is caused by mechanical unloading of muscle and this leads to reduced muscle mass without fiber attrition. Skeletal muscle stem cells (satellite cells) and myonuclei are integrally involved in skeletal muscle responses to environmental changes that induce atrophy. Myonuclear domain size is influenced differently in fast and slow twitch muscle, but also by different models of muscle wasting, a factor that is not yet understood. Although the myonuclear domain is 3-dimensional this is rarely considered. Apoptosis as a mechanism for myonuclear loss with atrophy is controversial, whereas cell death of satellite cells has not been considered. Molecular signals such as myostatin/SMAD pathway, MAFbx, and MuRF1 E3 ligases of the ubiquitin proteasome pathway and IGF1-AKT-mTOR pathway are 3 distinctly different contributors to skeletal muscle protein adaptation to disuse. Molecular signaling pathways activated in muscle fibers by disuse are rarely considered within satellite cells themselves despite similar exposure to unloading or low mechanical load. These molecular pathways interact with each other during atrophy and also when various interventions are applied that could alleviate atrophy. Re-applying mechanical load is an obvious method to restore muscle mass, however how nutrient supplementation (e.g., amino acids) may further enhance recovery (or reduce atrophy despite unloading or ageing) is currently of great interest. Satellite cells are particularly responsive to myostatin and to growth factors. Recently, the hibernating squirrel has been identified as an innovative model to study resistance to atrophy.

## Introduction

Skeletal muscle plays a significant role in quality of life and is essential for health and survival. It is highly organized at the micro- and macroscopic level and plays a major role in mobility of the human body. Skeletal muscle accounts for ~40% of body mass, permits precise movements and is highly adaptive. The characteristic of plasticity allows skeletal muscle to change and adapt depending on the stimuli placed upon it. Increases in mechanical load and increasing workload will stimulate muscle hypertrophy, while removal of mechanical load will lead to muscle atrophy as an appropriate adaptation to hypogravity (Goldberg et al., 1975).

Skeletal muscle has the uncanny ability to alter its phenotype depending on the mechanical load placed upon it. Disuse is an expansive label for the low mechanical load or mechanical unloading of muscle; with the most severe example being that of lack of gravity in spaceflight. The ground-based model, bed rest, simulates spaceflight. Physiological changes during bed rest include 6–24% reduction in muscle mass and strength (Narici and de Boer, 2011) and other disuse associated changes in skeletal muscle (Brooks et al., 2008).

Morphological changes with atrophy include a decreased cross-sectional area of muscle fibers, concomitant reduced whole muscle volume and mass, but no decrease in number of fibers (Nicks et al., 1989). This is different to age-related muscle fiber atrophy which is accompanied by a reduction in number of muscle fibers (Lexell et al., 1988). The process of muscle atrophy is highly regulated and results in reduced protein content, reduced force production, increased fatigability and decreased insulin sensitivity (Fauteck and Kandarian, 1995; Harrison et al., 2003), decreased capillary density of both fiber types and disruption of the 3-dimensional architecture of skeletal muscle (Hikida et al., 1997). Skeletal muscle also undergoes a shift in contractile capacity of the fibers toward fast glycolytic phenotypes (Fauteck and Kandarian, 1995; Fitts et al., 2000). For example, the soleus muscle which is predominantly composed of slow twitch fibers is a postural muscle and highly susceptible to disuse and fiber type switching (Booth and Baldwin, 1996).

Disease-induced atrophy (cachexia) is seen in disease states such as cancer, AIDS, renal failure, congestive heart failure, chronic obstructive pulmonary disease (COPD) and burns. In addition to disuse atrophy, cachexia involves an intricate cytokine and inflammatory response inducing signaling cascades and gene transcription. This review will focus on disuse atrophy and selected growth factor-related signaling. Human models of disuse, such as immobilization and sedentary lifestyle (inactivity) include decreased mechanical loading. Immobilization can occur as a result of various injuries, mostly not of skeletal muscle so the influence on skeletal muscle is considered a side effect. Immobilization can also be used as a model to investigate disuse and load reduction on skeletal muscle as a research intervention. Unilateral lower limb suspension involves suspension of one limb while the other is used for movement assisted by crutches. This model resulted in 5–10% reduction in CSA of Quadriceps muscle after 4 weeks (for more detailed review about ULLS, the reader is referred to Hackney and Ploutz-Snyder, 2012). Immobilization (cast or leg brace) in humans can lead to ~12% decrease in leg mass (for more detailed review about immobilization, the reader is referred to Marimuthu et al., 2011).

Skeletal muscle unloading in rodents is best modeled by hindlimb suspension (HS; Morey-Holton and Globus, 2002), which also leads to significant skeletal muscle atrophy particularly in postural, slow-twitch muscles, such as the soleus muscle (Templeton et al., 1984; Fitts et al., 1986; Riley et al., 1987). The HS model involves the animal being suspended by the tail which prevents the back legs from bearing any weight whilst the animals support their weight on the front legs. The front legs do not take on the entire weight of the animal since some of the loading is compensated for by the pulley and wire system that allows the animal to still move freely in the cage. For a recent extensive review on disuse atrophy and unloading models, the reader is directed to Baldwin et al. (2013). Of particular interest are the alterations in MHC and fiber shifting away from slow-oxidative fiber type toward fast glycolytic fiber type in the soleus muscle with HS (Templeton et al., 1984; Elder and McComas, 1987; Riley et al., 1987; Tsika et al., 1987). This observation is consistent regardless of the duration of unloading in different studies. Because of the location of the MHC genes this suggests that the basal gene expression by the myonuclei is altered. Thus a concept not frequently considered arises: constitutive gene expression is altered once the adaptive phase is complete. To understand the role of the myonuclear response to atrophy it is important to investigate myonuclei as entities in their own right. The fact that a muscle fiber is multinucleated and large adds to the complexity of the myonuclear responses. On the other hand the muscle precurser cells (satellite cells; SCs) are mononuclear and typically quiescent, hence their response to disuse may not be similar to those of the myonuclei.

There is a plethora of research which has been carried out in human subjects with differing methods and timescales of atrophy-inducing stimulation. Two recent studies from van Loon's laboratory demonstrate that after 5 days cast immobilization (Dirks et al., 2014) and 14 days cast immobilization (Wall et al., 2013) there are significant losses in muscle strength and size. Five days of immobilization led to 3.5% reduction in quadriceps CSA and 9% muscle strength (Dirks et al., 2014). In young males, there was a 8% decrease in muscle cross-sectional area and a 23% reduction in quadriceps strength after 14 days immobilization (Wall et al., 2013). In addition, data from the Kjaer laboratory also report decreases in fiber size (10; 20%) and strength (13; 20%) after 4 and 14 days, respectively (Suetta et al., 2012, 2013). The decrease in whole muscle strength is also reflected in reduced specific force of single muscle fibers (Hvid et al., 2013). Bed rest studies of longer duration (e.g., 28 days) consistently show reduced muscle strength and size (Hikida et al., 1989; LeBlanc et al., 1992; Edgerton et al., 1995; Ferrando et al., 1996; Bloomfield, 1997; Brooks et al., 2008). In skeletal muscle from individuals who were 9 years post-spinal cord injury, there was considerable atrophy compared to controls and 90% of the fibers were type II fibers (Verdijk et al., 2012).

Skeletal muscle is a key tissue in maintaining functional ability and contributing to health status. The aim of this review is to highlight the important and integrative role of SCs and myonuclei in skeletal muscle homeostasis with the focus particularly on atrophy. Understanding the role of myonuclei and SCs both in load-bearing and a variety of disuse conditions may lead to therapies to combat deleterious alterations in skeletal muscle that are specifically targeted to these progenitor cells. Since both SCs and myonuclei respond to environmental changes, the involvement of SCs in skeletal muscle health as well as for skeletal muscle therapies should be further investigated.

## Myonuclei and myonuclear domain

Despite being post-mitotic and unable to divide and replicate, myonuclei remain essential for skeletal muscle homeostasis, maintenance and adaptive responses. Therefore analysis of myonuclei may shed light on mechanisms responsible for muscle fiber loss or on responses to interventions to fight physical disability such as that seen with ageing and disease (Malatesta and Meola, 2010).

The theory of myonuclear domain suggests that each skeletal muscle nucleus governs an area of surrounding cytoplasm (Hall and Ralston, 1989) and produces enough protein to support the limited area of cytoplasmic and structural proteins within the local “domain” (Pavlath et al., 1989). Research has consistently reported that in situations of positive change in fiber size, i.e., with muscle growth, hypertrophy or overload, there are increases in myonuclear number as the fiber increases in size (Allen et al., 1995; McCall et al., 1998; Roy et al., 1999a; Adams et al., 2002; Petrella et al., 2006; VanderMeer et al., 2011). However, a somewhat flexible myonuclear domain size is likely especially during e.g., the early phase of an adaptive response (Kadi et al., 2004), either increase or decrease in fiber size or change in metabolic status.

Myonuclear domain size is different between fast and slow fiber types (Burleigh, 1977). Slow muscle fibers have higher rates of protein turnover (Booth and Thomason, 1991) and higher oxidative capacity has been associated with a higher level of protein synthesis (Roy et al., 1999b). In general, slow fibers have larger numbers of myonuclei (Edgerton and Roy, 1991) which leads to a smaller calculated myonuclear domain size (Tseng et al., 1994; Allen et al., 1995; Hikida et al., 1997; Brooks et al., 2009). Fast muscle fibers have lower oxidative capacity, relatively lower numbers of myonuclei and larger myonuclear domain sizes (Burleigh, 1977; Tseng et al., 1994). However, it is well known that fast twitch fiber sub-types have widely varying oxidative capacity and multiple hybrid isoforms (Kohn et al., 2007). It has also been reported that there is a positive correlation between myonuclear number and fiber size in young mice and this relationship is lost during adulthood (Bruusgaard et al., 2006). More research needs to be done to unravel the fiber type/oxidative capacity and age and myonuclear domain relationship.

## Myonuclear domain and disuse atrophy

While the myonuclear domain theory is well substantiated for the increase and incorporation of myonuclei with muscle hypertrophy (see above), the response of the myonuclear number and myonuclear domain size with atrophy is less conclusive. Despite less being known about the influence of disuse on myonuclei, the alterations in muscle morphology, biochemistry and physiology suggest that the myonuclei are influencing features of change within the muscle as well as being influenced by these alterations. Furthermore, atrophy from disuse occurs rapidly and its development is not simply the reverse of skeletal muscle hypertrophy. Myonuclear loss may follow a different timescale, occurring at a slower pace than the loss in muscle fiber size (VanderMeer et al., 2011). This may further complicate our understanding of change vs. no change of myonuclear domain size in response to an intervention. It is therefore important to distinguish between the adaptive phase and the new steady state when considering the muscle response to unloading.

Based on the myonuclear domain theory and the response to muscle growth, it could perhaps be inferred that with muscle atrophy there would be an accompanying loss of myonuclei resulting in maintenance of the myonuclear domain size (Siu and Alway, 2009). This inference relies on acceptance of the myonuclear domain theory that hypothesizes that each myonucleus can control only a certain domain for protein synthesis and general “maintenance.” Although this hypothesis was based on solid observational data, the mechanisms controlling the removal of nuclei under conditions that cell death (myofiber necrosis) is absent, is far from understood. Nonetheless, decreased myonuclear numbers are noted in muscles undergoing a variety of atrophy-inducing experimental conditions in humans and animals such as spinal cord isolation and transection, microgravity, hind limb suspension and chronic denervation (Darr and Schultz, 1989; Schmalbruch et al., 1991; Allen et al., 1995, 1996, 1997b, 1999; Day et al., 1995; Rodrigues Ade and Schmalbruch, 1995; Hikida et al., 1997). However, there have also been recent reports to suggest that atrophy with HS does not lead to loss of myonuclei (Bruusgaard et al., 2012).

Myonuclei can be removed by a number of different processes, mainly apoptosis or autophagy. Apoptosis is a tightly regulated process of highly coordinated “programmed cell death.” The process of apoptosis is a vital mechanism to allow normal development, tissue turnover and immunological function (Thompson, 1995) and to maintain tissue homeostasis throughout the lifespan. Apoptosis as a means of cell death is easy to conceptualize for mononuclear cells or cells with low nuclear number. However, in a multi-dimensional, multinucleated cell, how and why are particular nuclei targeted and not adjacent nuclei? There appears to be a fiber type-specific response of myonuclear loss, with a greater decrease in myonuclear number in Type I (Zhong et al., 2005) fibers that respond to unloading with more severe atrophy. In contrast excess glucocorticoid production such as that with cachexia results in fast glycolytic fibers being more affected (recently reviewed by Schakman et al., 2013). This further highlights the specificity and complexity inherent in regulation of disuse atrophy and by extension the control of myonuclear domain.

Regardless of the unknown mechanisms initiating myonuclear loss, elimination of myonuclei by nuclear apoptosis remains a rational idea to explain myonuclear domain size consistency in the face of atrophy, reviewed by Siu and Alway (2009). Allen et al. (1997a) published the first research demonstrating that the myonuclei lost with atrophy (HS) were eliminated by “programmed nuclear death,” apoptosis. Myonuclear apoptosis has been identified with muscle disuse/unloading/wasting and can be measured with the terminal deoxynucleotidyl transferase-mediated deoxyuridine triphosphate nick end-labeling (TUNEL) or by DNA fragmentation in gel electrophoresis (Allen et al., 1997a; Dupont-Versteegden et al., 1999, 2006; Smith et al., 2000; Leeuwenburgh et al., 2005). For example, after 16 days of HS, rat soleus muscle had reduced number of myonuclei and the size of the myonuclei were increased after HS but the DNA content did not differ (Wang et al., 2006). However, recent advances in techniques for measuring apoptosis, particularly the ability to measure *in vivo* and to differentiate between nuclear loss inside and outside the muscle cell have permitted more accurate assessment of apoptosis in the muscle. In a number of recent publications, small increases in TUNEL+ nuclei have been reported but the location of these nuclei was outside the sarcolemma and thus they have been identified as stroma cells (Bruusgaard et al., 2012; Suetta et al., 2012).

In light of the above discussion, the concept of muscle atrophy coinciding with loss of myonuclei to maintain myonuclear domain size is not conclusive. Nutritional restriction has been reported to reduce muscle fiber size but not myonuclear number thus decreasing the myonuclear domain size (Winick and Noble, 1966; Pitts, 1986). Some studies have found no loss of myonuclei or myonuclear apoptosis with situations of atrophy (Wada et al., 2002; Gundersen and Bruusgaard, 2008). As highlighted in an excellent review on myonuclear domains and muscle atrophy (Gundersen and Bruusgaard, 2008), a lot of the research findings noting myonuclear loss are based on cross-sectional histological assessment of myonuclei at a specific time point. Bruusgaard and Gundersen (2008) conducted an elegant study measuring *in vivo* time lapse of single fibers and found that after 4 weeks of denervation there was a 50% decrease in size of muscle fiber and no change in myonuclear number. Other studies using single fiber analysis have also found no change in myonuclear number in rodent muscle (Wada et al., 2002; Aravamudan et al., 2006).

The method used to measure myonuclei and myonuclear domain size is also important to consider for careful interpretation. Cell culture techniques provide a means for easy myonuclear analysis after intervention. However, the number of myonuclei available for analysis is high and the proportion of myonuclei for a given volume of myotube is more prominent than in whole muscle where the contractile protein content overwhelms the internal content of the fiber. The timing of the myonuclear sampling is also important when determining whether apoptosis actually occurs and it is difficult to maintain myotubes in culture for long periods. Myotubes in culture are also often not subjected to contractile forces and the relevance to disuse atrophy might be questioned, although this does not preclude the investigation of atrophy induced by other methods.

Loading of muscle prior to the induction of atrophy may also influence the acute responses, especially to unloading. Siu et al. (2005) reported that in recently hypertrophied muscle (with increase in myonuclei) myonuclei undergo apoptosis during disuse. Bruusgaard et al. (2010) investigated this further by investigating the concept of “muscle memory” by overloading EDL muscle of mice and rats to stimulate hypertrophy. The EDL muscle was then denervated to induce muscle atrophy. In contrast to Siu et al.'s work, this model of overload led to myonuclear accretion with hypertrophy, and the new myonuclei persisted even with atrophy after denervation (Bruusgaard et al., 2010). This maintenance of myonuclei in the animals with overload and denervation was not seen in the animals who underwent only denervation. The authors suggest that previous hypertrophy bouts may protect skeletal muscle against myonuclear loss with atrophy. This may be of benefit for individuals who will undergo bouts of enforced disuse such as that seen with immobilization and/or bed rest following elective surgical procedures.

It is also important to highlight that the various experimental models used to investigate disuse atrophy differ substantially and hence have varying degrees of physiological application. Although animal models may seem too different from human models, it is of extreme importance for development in the area that both human and animal models are included in the research, whilst still keeping in mind that these choices may differ in the extent that the intervention can be applied. The most typical models of disuse atrophy are: denervation or hindlimb suspension in rodents; immobilization or bed rest in humans. Previously, the techniques used for analysis included histology, immunohistochemistry, gene expression and protein content, but *in vivo* assessment, single fiber assessment, myonuclear assessment, myonuclear domain size estimations were typically lacking in most studies. Further, the timing of muscle or myotube sampling will influence the response recorded. As discussed in this review, this is particularly evident when assessing the SC count in humans after short immobilization bouts, longer bed rest and/or longer disuse seen with spinal cord injury (Brooks et al., 2010; Verdijk et al., 2012; Snijders et al., 2014). The muscle chosen for analysis i.e., slow twitch soleus muscle, or fast twitch EDL muscle, or mixed muscle from humans, are key factors in unraveling the complex but enticing role of myonuclei in skeletal muscle atrophic situations. Recent human research has highlighted sex differences in skeletal muscle response to immobilization (Yasuda et al., 2005) which should also be taken into consideration. Finally, new methods should be developed or taken up by more researchers, for example the 3-dimensional analysis of myonuclear domain size and muscle volume measurements.

A fundamental question related to myonuclei, myonuclear domain and the response to atrophic situations is the involvement of skeletal muscle stem cells (SCs) in the maintenance of muscle mass, and the response to muscle loss.

## Satellite cells

Myonuclei in skeletal muscle are post-mitotic and cannot replicate. Therefore, any increase in myonuclear number such as required for growth and repair is a result of SC fusion, although also to a very minor extent other stem- or stem cell-like cells (Boppart et al., 2013). The quantity of SCs differs between muscles, fiber types, developmental stages and species. In general, there are more SCs in type I fibers than type II fibers; the number of SC number does not change from birth to adulthood despite increases in fiber size (Verdijk et al., 2013). However, this is age, species and muscle specific (Gibson and Schultz, 1982; Mackey et al., 2009; Verdijk et al., 2013). SCs are responsive to various environmental influences, including disuse.

SCs have distinctive morphological features, including large nuclear-to-cytoplasmic ratio, few organelles, small nuclei and condensed interphase chromatin clearly visible using electron microscopy (Mauro, 1961). While the gold standard measurement of SCs is with electron-microscopy, SCs can also be identified with light microscopy and immunohistochemical labeling of factors expressed by the SCs (for further details on transcription factors and other markers expressed by SCs see the recent review by Yin et al., 2013). While SCs express these markers, labeling with some of these will also identify other cells. Where this is the case, the marker should not be used alone to identify SCs but rather in combination with another or several other known markers that (i) label the SCs but not the alternate cell; (ii) label the alternate cell but not the SCs; or (iii) label adjacent structures such as the sarcolemma or basal lamina. The choices for labeling with immunohistochemistry should be carefully considered, particularly when one is assessing SCs after an unusual intervention such as disuse that may affect SCs and myonuclei substantially.

Under normal situations SCs remain quiescent (Bischoff, 1990). Upon activation in response to stimuli present during injury or growth, SCs enter the cell cycle (Bischoff, 1990). When activated, SCs proliferate and express myogenic regulatory factors (MRFs), MyoD and Myf5 (Yablonka-Reuveni and Rivera, 1994; Zammit et al., 2002). After proliferation, most cells maintain MyoD but downregulate Pax7 and commit to differentiation via activation of myogenin. Other myoblasts maintain Pax7 but down-regulate MyoD and withdraw from the cell cycle regaining markers that characterize quiescence (Nagata et al., 2006; Day et al., 2007). All SCs undergo a stage of co-expressing Pax7 and MyoD before the decision to self-renew or differentiate is made. SCs have the ability to undergo both asymmetric as well as symmetric divisions (Kuang et al., 2007) either producing identical progeny or different progeny (Kuang et al., 2007).

Over recent years, a number of key publications have demonstrated that SCs are not all the same (Zammit, 2008; Biressi and Rando, 2010; Scharner and Zammit, 2011) and variations exist even between SCs in the same muscle (Ono et al., 2012). It is thought that the surrounding area (niche) of the SC plays a role in the fate and adaptive responsiveness of the SC (Kuang et al., 2007). Therefore, the environment that influences muscle fibers and myonuclei will also have an effect on SCs. The role of SCs in skeletal muscle atrophy and recovery is less well characterized than their role in growth and injury repair. However, any reduction in the ability of SCs to respond to injury and trauma during atrophy or upon reloading will be a further detrimental episode for the muscle (Chargé and Rudnicki, 2004).

Although this review is focused on muscle atrophy, it is important to highlight that recent research has questioned the absolute requirement of SCs for muscle hypertrophy. Skeletal muscle can respond to hypertrophy stimulation even in animals without SCs. In adult Pax7-DTA mice, which have greater than 90% of SCs removed, the muscle can respond to an overload stimulus (McCarthy et al., 2011). These muscles without SC have a blunted response to regeneration, but are still able to hypertrophy (McCarthy et al., 2011). A number of studies in animals have reported skeletal muscle hypertrophy without SC activation or incorporation of myonuclei (Amthor et al., 2009; Blaauw et al., 2009; Raffaello et al., 2010; Lee et al., 2012; Wang and McPherron, 2012). These studies demonstrate that hypertrophy can occur independently of both SC proliferation as well as myonuclear accretion. This highlights the ability of rodent skeletal muscle to respond to overexpression induced hypertrophy (i.e., AKT overexpression—Blaauw et al., 2009), myostatin knock-out induced hypertrophy (Amthor et al., 2009), hypertrophy with overexpression of JunB in cell culture (Raffaello et al., 2010). Further, hypertrophy induced by myostatin inhibition in adult mice preceded incorporation of myonuclei (Wang and McPherron, 2012). This is also reported in animals which are transgenically modified to inhibit syndecan4 or Pax7, there is a hypertrophy response to hypertrophy stimulated by myostatin blockade, even without SC proliferation and fusion (Lee et al., 2012).

## Satellite cells and disuse atrophy

Early studies indicated that atrophic conditions lead to increases in the number of apoptotic myonuclei both inside and outside myofibers (Allen et al., 1997a; Vescovo et al., 1998). However, more recent studies indicate no change in myonuclear numbers with atrophy, and loss of SCs with atrophy is not a consistent finding. For example, in recent human studies of 14 days immobilization (Snijders et al., 2014), 28 days bed rest (Brooks et al., 2010) there appears to be no change in SC number, but Suetta et al. (Suetta et al., 2013) report an increase in SCs after 14 days immobilization. In contrast, in a recently published paper reporting SC numbers in states of severe disuse atrophy, spinal cord injury, individuals had significantly lower SC numbers in both type I and type II fibers (Verdijk et al., 2012). Thus, the severity of disuse induced atrophy and the duration of the condition must be considered when interpreting the existing literature.

Whether or not some SCs are lost during atrophy, it is still an open question how and why function (e.g., mitotic ability) of remaining SCs are is altered during atrophy and if so, whether their functionality is can be restored with reloading. In studies investigating alterations in SC content and proliferative ability, the age and growth stage of the animals is important. To investigate the role of SCs and the effect which atrophy has on their function, using young animals whose growth has not stabilized can shed light on factors not investigated in other models. For example, the SC may be more responsive during growth and this may alter their response to the atrophy-inducing intervention (see Darr and Schultz, 1989). SC response to potential interventions to reduce atrophy may also be different when comparing the growing, the adult or aged animal, thus interpretation and application should be done with care. Mechanical unloading appears to reduce the number of SCs (Darr and Schultz, 1989; Mozdziak et al., 1998; Matsuba et al., 2009). This may be due to apoptosis of SCs as well as myonuclei in situations of atrophy (Jejurikar et al., 2002; Jejurikar and Kuzon, 2003; Ferreira et al., 2006). Wang et al. (2006) investigated the mechanisms underlying the SC response to HS, particularly the distribution of SCs and the level of mechanical load applied to the muscle. Their results suggest that the regulation of SCs is dependent on the mechanical loading and the location of the SC along the muscle fiber. Unloading resulted in a significant decrease in SC number, particularly at the central region of the muscle fibers (Wang et al., 2006).

In contrast, Darr and Schultz (1989) showed that the alterations in myonuclear and SC population were dependent on the time course of HS. They investigated 30 days of HS in rat muscle, particularly fast-twitch extensor digitorum longus (EDL) and slow-twitch soleus muscle. After 3 days of HS, the authors report a reduction in SC number and halting of mitotic activity in both soleus and EDL muscle. Between 3 and 10 days of HS the SCs began dividing and there was an increase throughout the remainder of the HS. In addition to the increase in dividing cells, the EDL muscle also appeared to have a compensation response with 4x increase in active SCs in EDL myofibers. Although this study found that the earlier response was reduced SC number and mitotic activity, Ferreira et al. (2006) reported an intense peak of proliferating activity in SCs after 6 h of HS (SC duplication) followed by an increase in myonuclei at 12 h of HS (Ferreira et al., 2006). During this time there also appeared to be apoptosis (Ferreira et al., 2006). Investigating the morphometric and ultrastructural properties of SCs in rat soleus during immobilization, Kujawa et al. (2005) found caveolae on the SCs and a decrease in SC activity. Clearly, the SC response to atrophy is biologically complicated. Interestingly, in a human study, when specifically labeled with Pax7 and TUNEL+, there were no apoptotically marked SCs after 14 days of immobilization (Suetta et al., 2012).

In a seminal set of experiments, Mitchell and Pavlath examined the properties of muscle precursor cells (MPCs include both SCs and other stem-like cells between myofibers, fat and in blood vessels) after 14 days of HS in mice (Mitchell and Pavlath, 2004) and subsequent recovery. HS led to decreased number of MPCs and these MPCs taken from atrophied muscle cells could not proliferate and differentiate *in vitro* into normal myonucleated myotubes. Recovery appeared to lead to reversal of the dysfunctional properties highlighting that these changes are transient, lasting only until normal weight bearing activities are resumed. This would seem to imply that SC reactivation is integral to recovery. However, in young animals Jackson et al. (2012) concluded that SCs are not required for muscle growth following atrophy since restoration of myonuclear domain size with reloading after HS was independent of SCs.

Mozdziak et al. (2001) investigated the interaction between injury and HS, specifically the myofiber size, SC mitotic activity and DNA unit size after resumption of normal activities in soleus muscles of rats. They found muscle injury combined with inactivity (HS) caused long-term reduction in muscle size compared with injury in weight bearing animals. After HS the SCs responded to compensate for the muscle injury but this was not enough to return myonuclear numbers to levels similar to that of the animals who had not undergone HS (Mozdziak et al., 2001). The authors speculated that the HS caused a disruption in “DNA expression unit size” (such as seen in Mozdziak et al., 2000) and combined with the reduction in SC mitotic activity results in a reduced size of soleus muscle even after 9 weeks of reloading. This interpretation indicates that the myonuclear domain size is not necessarily primarily responsive to changes in muscle size, but may itself have an active influence on muscle size.

Interestingly, while the number of SCs does not appear to change in human muscle with short-term disuse (28 days or less), it should be highlighted that alterations in MRFs have been documented. During 14 days of immobilization, Snijders et al. (2014) report myogenin mRNA expression doubled. Further, after 28 days bed rest and essential AA supplementation without exercise, MyoD transcripts were elevated after 28 days and remained elevated after recovery (Brooks et al., 2010). Thus, SCs are not quiescent when the environment is changing and there may be a myogenic response occurring despite muscle proteolysis.

Investigations into treatments to reduce atrophic response during HS have found that application of low-frequency electrical stimulation (LFES) partially rescues the loss of SCs and lessens the reduction in muscle cross-sectional area (Zhang et al., 2010); rescues SCs and maintains their viability for muscle regeneration (Guo et al., 2012) and reduces loss of myonuclear domain size (Zhang et al., 2010). Zhang et al. (2010) applied LFES during HS in a rat model and investigated SC response in soleus and EDL muscles. The authors reported evidence of a reduced capacity for SC activation, proliferation and differentiation in soleus muscle after 28 days HS, findings that were partially attenuated by LFES. Interestingly, there was no atrophy in the EDL muscle and no alteration in myonuclear domain size in the EDL muscle (Zhang et al., 2010).

In summary, the balance of data indicates that in animals SC numbers decrease while in human studies SC numbers remain similar or even increase unless the atrophic environment is particularly severe. Furthermore it seems the regenerative capacity, and indeed the myogenic regulation of the remaining SC is maintained. Atrophy occurs under many conditions and unraveling how SCs respond in the various conditions will need to include better examination of the niche environments in which the SCs reside and not only the quantitative responses. The response of SCs to disuse atrophy is further complicated by the time course of exposure to the condition and therefore research should be including a broader timescale without sacrificing investigation of the rapid early responses. Investigation of early responses should include cell signaling events within SC and not only muscle tissue itself. Extensive research has been done on the molecular pathways which influence atrophy (see below) and some evidence exists that SC are also influenced significantly by these pathways.

## Molecular signaling pathways

The molecular mechanisms underpinning muscle atrophy with disuse remain to be fully elucidated. The next section aims to describe 3 of the key molecular pathways which are linked to skeletal muscle atrophy: Atrogin-1/MAFbx and MuRF1; the IGF-1-AKT-mTOR pathway; and the Myostatin Pathway. Existing authoritative review articles will be highlighted. Further, we aim to discuss how these pathways are related to SCs and myonuclear responses to atrophy.

## Ubiquitin proteasome pathway (MAFbx/MuRF1) and disuse atrophy

The ATP-dependent ubiquitin proteasome pathway is the primary degradation pathway of skeletal muscle in response to inactivity and disuse. The ubiquitin-proteasome pathway is involved in breakdown of short-lived proteins or long-lived myofibrillar proteins in skeletal muscle. Three distinct components are required for muscle breakdown using the ubiquitin proteasome pathway. E1 ligases which activate ubiquitin, E2 ligases that are responsible for transferring the activated ubiquitin to the protein molecule that is then targeted for degradation and the E3 ligases which regulate the actual transfer of ubiquitin to the protein. Two important skeletal muscle specific ubiquitin E3 ligases are Muscle-specific RING Finger protein1 (MuRF1) and Muscle Atrophy F-box (MAFbx/atrogin-1).

MAFbx and MuRF1 are both primarily expressed in skeletal muscle and are upregulated in several models of disuse (Bodine et al., 2001). MAFbx and MuRF1 were first identified following profiling in mouse atrophy after fasting and immobilization in a profound set of experiments published by both Bodine et al. (2001) and Gomes et al. (2001). In knock-out models, animals which cannot make MAFbx or MuRF1 proteins appear to be similar to the wild-type animals with phenotypically similar muscle and normal body weight (Bodine et al., 2001). However, MAFbx knockout mice had a reduced loss of muscle mass (56% sparing) after 7 and 14 days (Bodine et al., 2001). MuRF1 knockout mice had a 36% sparing of muscle compared to wild-type mice 14 days after denervation.

Both MAFbx and MuRF1 appear to be early markers of disuse atrophy. MAFbx and MuRF1 mRNA levels are rapidly increased in numerous models of atrophy and are thought to contribute to the initiation of the atrophy process (Foletta et al., 2011). They are increased after spaceflight in rodents (Allen et al., 2009), after 3 days of ULLS in humans (Gustafsson et al., 2010); and after immobilization (Jones et al., 2004; Abadi et al., 2009). MAFbx and MuRF1 are regulated by the family of Forkhead box O (FOXO) transcription factors (Stitt et al., 2004). FOXO is a family of transcription factors that are involved in metabolism, apoptosis and cell cycle progression (Carlsson and Mahlapuu, 2002). When FOXO transcription factors are dephosphorylated they enter the nucleus and act to suppress growth and promote apoptosis (Ramaswamy et al., 2002). AKT (also called protein kinase B or PKB) phosphorylates FOXO transcription factors on multiple sites leading to their exclusion from the nucleus. Under normal physiological conditions, AKT thus inhibits the transcriptional functions of FOXO and FOXO is unable to suppress growth or upregulate atrophy and myonuclei are maintained.

## IGF-1-PI3K-AKT-mTOR and disuse atrophy

Extensive literature supports the role of the IGF-1-PI3K-AKT-mTOR pathway in regulation of skeletal muscle hypertrophy (for example see reviews Glass, 2003; Schiaffino et al., 2013). Activation of this pathway leads to increases in translation initiation factors ultimately leading to increased protein synthesis: IGF1 activates Phosphatidylinositol 3 kinase (PI3K), which phosphorylates phosphatidylinositol-4,5-bisphosphate to phosphatidylinositol-3,4,5-trisphosphate in the membrane; this creates a binding site for AKT; activation of AKT phosphorylates and activates mammalian target of rapamycin (mTOR) kinase; mTOR increases protein synthesis by phosphorylation and activation of p70S6kinase and eukaryotic translation initiation factor 4E binding protein 1, both of which are involved in translation and protein synthesis. The activation of AKT appears to be a crucial determinant of the cellular signaling processes and appears to sit at the transition point between atrophy and hypertrophy (again, see Glass, 2003; Schiaffino et al., 2013 for review).

With situations of disuse, AKT is not activated and this contributes to muscle atrophy via FOXO (Sandri et al., 2004). Animals with overexpression of FOXO have reduced muscle mass and this appears to be related to increases in MAFbx and MuRF1 (see above). Increased FOXO transcript levels in rodents were reported after spaceflight (Allen et al., 2009) and decreased phosphorylation of AKT levels were reported after 10 days HS (Sugiura et al., 2005). Alterations in the IGF1-AKT-mTOR pathway are linked directly to alterations in the ubiquitin proteasome ligases, MAFbx and MuRF1 (Kandarian and Jackman, 2006). Activation of the IGF-1 pathway is significant in reducing FOXO translocation. Inhibition of the IGF-1 pathway caused FOXO translocation to the nucleus, promoted growth suppression and stimulated proteolysis (Stitt et al., 2004). In humans, after 5 days of immobilization without intervention, transcript levels of MAFbx and MuRF1 were increased (Dirks et al., 2014). However, no increases in FOXO were noted after 4 or 14 days of immobilization (Suetta et al., 2012).

Relocation of FOXO to the nucleus also activates genes involved in cell death and cell cycle inhibition (Stitt et al., 2004) and may therefore potentially affect SC proliferation. IGF-1 is known to act directly on SCs. IGF-1 leads to SC proliferation and its absence is associated with lower proliferation capacity. Therefore, interventions that focus on the IGF-1/mTOR pathway will also induce activation of the support-system for addition of myonuclei. IGF-1 acts both intracellularly and extracellularly to induce both proliferation and differentiation of SCs (Bischoff, 1986; Adams and Haddad, 1996; Adams, 1998; Cameron-Smith, 2002; Mourkioti and Rosenthal, 2005) and different isoforms of IGF-1 may be responsible for these (see Review for further reading of the IGF-1 and skeletal muscle regeneration and hypertrophy: Philippou et al., 2007). mTOR has been described as the master regulator of cellular processes and has been linked to differentiation in C2C12 myobalsts (Erbay et al., 2003; Han et al., 2008). While its role in myogenic differentiation has not been conclusively clarified, mTOR is thought to play a role in regulating MyoD stability (Sun et al., 2010). Further clarification of the interaction of mTOR, as well as other key components of the pathway, with SC function remain to be elucidated.

## Myostatin and disuse atrophy

Myostatin (growth-differentiation factor 8, GDF8) is a member of the transforming growth factor (TGF)β superfamily and a negative regulator of muscle mass. Myostatin appears to be primarily found in muscle tissue (McPherron et al., 1997). It has been widely reported that either natural mutations or scientific knock-out animals without myostatin gene have hypertrophied muscles such as the “double muscled” cattle (Grobet et al., 1997; Kambadur et al., 1997; McPherron and Lee, 1997) and the significant hypertrophy in a child (Schuelke et al., 2004). Myostatin knockout mice were larger in size than wild-type littermates and had over 200% greater muscle mass (McPherron et al., 1997). When myostatin gene expression is blocked experimentally, there are 13–30% increases in skeletal muscle hypertrophy (Whittemore et al., 2003).

The relationship between myostatin and atrophy is less concrete. Overexpression of myostatin appears to lead to muscle atrophy in transgenic mice (Reisz-Porszasz et al., 2003). Increased levels of myostatin mRNA and protein levels are seen as early as 1 day after HS (Carlson et al., 1999), after sciatic nerve resection (Shao et al., 2007) and after 11 days of spaceflight in mice (Allen et al., 2009) and in humans after chronic disuse (Reardon et al., 2001). Serum myostatin levels were increased by 12% after 25 days of head down bed rest (Zachwieja et al., 1999) while myostatin transcript and protein levels were both increased after 3 days of ULLS (Gustafsson et al., 2010). Increased myostatin transcript levels have been report after 5 days (Dirks et al., 2014) while after 14 days of immobilization the same group report decreased protein levels of myostatin (Snijders et al., 2014).

Myostatin appears to inhibit muscle growth through inhibiting the AKT-mTOR pathway (less protein synthesis), upregulating the ubiquitin proteasomal pathway via FOXO (more protein breakdown) (McFarlane et al., 2006) and reducing SC differentiation (Langley et al., 2002; Zimmers et al., 2002). Myostatin activates withdrawal from the cell cycle in mammalian myoblasts and stimulates quiescence rather than differentiation or apoptosis (McFarlane et al., 2008) by inhibiting MyoD activity (Langley et al., 2002; McCroskery et al., 2003; Amthor et al., 2006; Manceau et al., 2008).

Promoting quiescence is not the only influence that myostatin has on SCs. It also seems to play a role in regulating SC self-renewal in cell culture (McFarlane et al., 2008). Excess myostatin inhibited Pax-7 expression, whereas inactivation of myostatin (by genetic inactivation or functional antagonism of myostatin) resulted in increased Pax7 expression (McFarlane et al., 2008). Increased myostatin protein levels have been linked with dysfunctional SCs in aging human muscle (McKay et al., 2012). While the intricate response of skeletal muscle to ageing is outside the scope of this review, this evidence suggests that increased myostatin levels seen with atrophy may contribute to the reduced proliferative ability of SCs and the reduction in the pool of parent SCs.

Myostatin activates the SMAD pathway and SMAD3 null mice had significant atrophy combined with increased levels of MuRF1 and decreased SC function (Ge et al., 2011). This response was initiated by increased myostatin levels in the SMAD3 null mice, and the response was abolished when myostatin was inactivated in these mice (Ge et al., 2011). SMAD3 may contribute to self-renewal of SCs. Myostatin also acts via other SMAD pathways and cell signaling downstream of myostatin interacts with the IGF-1 pathway.

Follistatin is a natural inhibitor of myostatin. Animals with overexpression of follistatin have increased muscle mass (Lee and McPherron, 2001; Haidet et al., 2008), and the muscles are larger than with myostatin knock-out alone (Lee, 2007). Overexpression of follistatin appears to result in increased SC activation as well as increased protein synthesis (Gilson et al., 2009). In this study, increased fiber size was accompanied by increases in myonuclear number (Gilson et al., 2009). The further study of the role of follistatin as a therapeutic aid in maintenance of muscle mass despite atrophy-stimulating conditions is certainly warranted.

SCs have the ability to differentiate across lineages including adipogenic and myogenic lineages. In situations of disuse, there are increased levels of intramuscular adipose tissue (IMAT). The increase in adipose may be due to the reduction of regenerative capacity of SCs (Chargé and Rudnicki, 2004) which may contribute to an abnormal shift toward the adipogenic lineage such as that seen in ageing skeletal muscle (Kirkland et al., 2002). SCs from obese animals produce myotubes that have impaired insulin sensitivity. In SCs cultured from obese animals there is an increased number progressing to the adipogenic lineage rather than the myogenic lineage which suggests that metabolic conditions lead to an increased proportion of SCs entering the adipogenic pathway which may contribute to the greater fat deposit in skeletal muscle (Scarda et al., 2010). It is thought that myostatin may be one of the key regulators of SCs and may play a role in determining the fate of SCs to adipogenic lineage or myogenic lineage (Deng et al., 2012).

In summary, despite atrophy occurring in the multinucleated muscle fibers, SCs are also influenced by the molecular pathways activated during the atrophy process. The alterations in both number and mitotic ability of SCs with atrophy are mediated, at least in part through increased myostatin. The influence of myostatin (and follistatin) on the potential of both SCs and pluripotent stem cells to differentiate into different lineages, is of extreme significance for skeletal muscle health as well as for prospects of clinical medicine and therapies aimed at maintaining and positively influencing skeletal muscle.

## Restoration of skeletal muscle after disuse atrophy

Reapplying mechanical load appears to be the most effective method to restore muscle mass, and increase myonuclear number, SC number and regenerative capacity.

Resistance exercise increases muscle mass and by increasing the load placed on the muscle which activates the PI3-AKT-mTOR pathway and increases protein synthesis. As little as one bout of resistance exercise in healthy individuals has been reported to increase IGF1 gene expression (Chesley et al., 1992). In young individuals, resistance exercise leads to increased protein synthesis after 2–4 h (Phillips et al., 1997) and this increase is maintained for 24–48 h in untrained individuals (Phillips et al., 1997).

In models of disuse, such as bed rest and immobilization, resistance exercise alone has been reported to reduce, but not completely alleviate muscle loss. Resistance exercise during 14 days of single leg immobilization in humans was sufficient to preserve quadriceps muscle mass (Oates et al., 2010) and during bed rest exercise alone reduces loss in muscle mass (Ferrando et al., 1997). As mentioned earlier, SCs appear to be activated and used with increases in mass from resistance exercise. In an interesting model to attempt to alleviate muscle loss with immobilization, during 5 days of HS, animals undertook resistance exercise with one leg (a combination of concentric, eccentric and isometric contractions) and the other leg remained as control. The leg which exercised during the HS maintained muscle mass and myofibril content (Adams et al., 2007). The stimulus was sufficient to increase gene expression of IGF1, myogenin and decrease myostatin. Further, increased p70S6K was reported. Thus, combination resistance exercise was sufficient to counter the initial alterations of disuse-induced muscle atrophy in mice (Adams et al., 2007).

Another form of atrophy prevention is electrical stimulation and stretching. However, these interventions did not reduce atrophy after 7 days following denervation in rats (Russo et al., 2010). This intervention reduced gene expression of MyoD, MAFbx, and MuRF1 levels whilst Myostatin gene expression was maintained, but there was no reduction in atrophy (Russo et al., 2010). Neuromuscular electrical stimulation has shown promising results in 5 days of immobilization in humans (Dirks et al., 2014). Applying electrical stimulation during 5 days of immobilization prevented the muscle loss and prevented the increase in myostatin, MAFbx and MuRF1 transcript levels (Dirks et al., 2014).

Protein synthesis and degradation are influenced by nutrient intake and intake of proteins and amino acids stimulate muscle protein synthesis and inhibits protein breakdown (Rennie et al., 1982). In particular, leucine, an essential amino acid, is a powerful stimulator of protein synthesis. Carbohydrate and protein are known to stimulate protein synthesis and can positively influence IGF1-mTOR-AKT pathway to stimulate protein synthesis and prevent upregulation of FOXO, MuRF1, and MAFbx; therefore nutritional intake could be a countermeasure in reducing muscle mass loss with disuse, particularly in situations where exercise is not feasible (such as hospitalized bed rest). Essential AA have consistently been shown to influence protein synthesis and alleviate some, but not all, of the loss of skeletal muscle experienced with bed rest (Paddon-Jones et al., 2004) but not to the same extent as exercise. However, with immobilization, amino acid supplementation does not appear to reduce loss of muscle mass (Stein and Blanc, 2011). An “anabolic resistance” was observed in healthy young individuals after immobilization (Glover et al., 2008) where there was decrease in muscle size and protein synthesis was 68% greater in the non-immobilized leg compared to the immobilized leg. There was also a reduced phosphorylation of AKT and p70S6K (Glover et al., 2008). Amino acid supplementation with immobilization improved protein synthesis but did not completely alleviate alterations with immobilization (Glover et al., 2008). Twenty-eight days of immobilization with protein and amino acid supplementation (28 g protein) did not prevent increases in myostatin, MuRF1 or MAFbx compared with immobilization alone (Bunn et al., 2011). In addition, after 14 days of immobilization there was a 31% decrease in post-prandial protein synthesis rate after consuming 20 g protein (Wall et al., 2013) demonstrating that the concept of anabolic resistance to protein ingestion occurs prior to 28 days. Studies assessing the influence of amino acid supplementation on SC and myonuclear response are an essential part of the keys to understanding the role of SCs and influence of exercise and nutrition.

Amino acid supplementation alone does not appear to reduce muscle atrophy, however combined with resistance exercise they provide an effective countermeasure. Some, but not complete, preservation of muscle mass and strength was reported after 28 days of bed rest with resistance exercise combined with amino acid supplementation (Brooks et al., 2008). Interestingly, the myogenic response was more pronounced in those who did not exercise during bed rest (receiving only the AA supplement) who had greater atrophy than those who exercised (Brooks et al., 2010). Myostatin transcript levels were increased significantly in the group who did not exercise compared with those who did. There was no difference in SC numbers after 28 days of bed rest or recovery indicating that the stimulus was not sufficient to increase and sustain SCs (Brooks et al., 2010). One can speculate that gravity combined with resistance exercise is needed to stimulate SC response.

Overexpression of IGF-1 did not protect mice against muscle loss with cast immobilization. After 1 week of reambulation after immobilization, mice with IGF-1 overexpression had enhanced muscle regeneration including increased muscle size, central myonuclei and Pax7+ cells (Stevens-Lapsley et al., 2010).

During rehabilitation exercise after disuse in humans, myostatin levels were supressed and sustained at lower levels throughout rehabilitation (Jones et al., 2004; Hittel et al., 2010). The reduced myostatin levels during rehabilitation exercise may act, at least in part, by releasing the inhibition on SCs and promoting muscle recovery via SCs (as discussed earlier) as well as increased AKT levels and activity (Morissette et al., 2009; Trendelenburg et al., 2009). These interventions are key to recovery from disuse atrophy and rehabilitation to functional status.

An excellent natural model to study resistance to atrophy is hibernation. Small mammals undergo long periods of reduced activity and hypocaloric intake. Hibernating animals are protected against muscle loss—despite inactivity and anorexia (for review, see Storey and Storey, 2007). Compared to the response of non-hibernating animals, such as humans and rodents, hibernating animals appear to be resistant to atrophy with disuse (Rourke et al., 2004a,b). Larger hibernating animals also appear to avoid protein loss and maintain functional capacity of skeletal muscle (Harlow et al., 2001; Lohuis et al., 2007). Hibernating animals have elevated MAFbx levels but this is not associated with atrophy (Rourke et al., 2004b). Myostatin protein levels are not increased during early hibernation and torpor, but increase during early arousal prior to resuming normal body temperature (Brooks et al., 2011).

In general, mammals lack the ability to prevent significant atrophy with disuse or disease. Hibernating grounds squirrels provide a natural model to study mechanism of resistance to atrophy in conditions of disuse and hypocaloric intake. The response of SCs and myonuclei could prove to be extremely insightful as to the natural response of muscle to resisting atrophy. By understanding the natural response to maintain myonuclear and SC function as well as with muscle atrophy will provide insight into the factors influencing atrophy and reduced function with disuse, normally an atrophy-inducing state.

## Summary

Since their first discovery in the 1960s, SCs have rightly played a prominent role in skeletal muscle research. Both SCs and myonuclei respond to the environmental changes which occur with disuse atrophy. The full role which they play to establish a new homeostatic environment for the muscle fiber, and/or the surrounding niche area, and the influence that the environment has on them, remains to be elucidated. The alterations in skeletal muscle with disuse atrophy such as myostatin and IGF1-AKT-mTOR appear to influence SCs as well as muscle mass. Despite no change in SC numbers with short duration human studies (<30 days), the SCs are not quiescent when their environment is changing but rather they are responding to the alterations in the niche area. This review brings together the current knowledge of myonuclear and SC response to disuse atrophy and highlights the complexity of the response in animals and humans.

### Conflict of interest statement

The authors declare that the research was conducted in the absence of any commercial or financial relationships that could be construed as a potential conflict of interest.
